# Ten Simple (Empirical) Rules for Writing Science

**DOI:** 10.1371/journal.pcbi.1004205

**Published:** 2015-04-30

**Authors:** Cody J. Weinberger, James A. Evans, Stefano Allesina

**Affiliations:** 1 Department of Ecology & Evolution, University of Chicago, Chicago, Illinois, United States of America; 2 Department of Sociology, University of Chicago, Chicago, Illinois, United States of America; 3 Computation Institute, University of Chicago, Chicago, Illinois, United States of America

*“…though a Philosopher need not be sollicitous that his style should delight its Reader with his Floridnesse*, *yet I think he may very well be allow’d to take a Care that it disgust not his Reader by its Flatness*, *especially when he does not so much deliver Experiments or explicate them*, *as make Reflections or Discourses on them; for on such Occasions he may be allow’d the liberty of recreating his Reader and himself*, *and manifesting that he declin’d the Ornaments of Language*, *not out of Necessity*, *but Discretion…”—Robert Boyle*, *Proëmial Essay* [1].

Scientists receive (and offer) much advice on how to write an effective paper that their colleagues will read, cite, and celebrate [2–15]. Fundamentally, the advice is similar to that given to journalists: keep the text short, simple, bold, and easy to understand. Many resources recommend the parsimonious use of adjectives and adverbs, the use of present tense, and a consistent style. Here we put this advice to the test, and measure the impact of certain features of academic writing on success, as proxied by citations.

The abstract epitomizes the scientific writing style, and many journals force their authors to follow a formula—including a very strict word-limit, a specific organization into paragraphs, and even the articulation of particular sentences and claims (e.g., “Here we show that…”).

For our analysis, we collected more than one million abstracts from eight disciplines, spanning 17 years. The disciplines were chosen so that biology was represented by three allied fields (Ecology, Evolution, and Genetics). We drew upon a wide range of comparison disciplines, namely Analytic Chemistry, Condensed Matter Physics, Geology, Mathematics, and Psychology (see table in S1 Text). We measured whether certain features of the abstract consistently led to more (or fewer) citations than expected, after accounting for other factors that certainly influence citations, such as article age (S1 Fig), number of authors and references, and the journal in which it was published.

We organized the most frequent suggestions into “Ten Simple Rules,” and probed them by testing a variety of features from the abstracts. Because the style and requirements for abstracts can vary dramatically between journals (S2 Fig), we normalized all the measures according to their distribution for each journal (S1 Text).

## Rule 1: Keep It Short

This is the most universally accepted piece of advice given to writers [3,7,9,11–13]. We tested this by examining the effect of shorter abstracts on citation, measuring the number of words (Rule 1a [R1a]) and number of sentences (R1b) in each abstract.

## Rule 2: Keep It Compact

The typical advice is to keep sentences or phrasing short, break compound sentences into simpler sentences, and remove any “unnecessary” words [2–6,9–12,14]. We evaluated this by measuring the effect of having sentences shorter than the mean for the journal where the article was published (R2).

## Rule 3: Keep It Simple

Canonical advice includes the prescription to use plain language and avoid jargon and technical terms [2–4,7,10,12,14]. Many of the most prominent journals state that their abstracts should be accessible to scientists working in different disciplines. To test this, we measured the proportion of words in the abstract that are found in a standard English dictionary (R3a) and that are present in a dictionary of “easy words” (R3b).

## Rule 4: Use the Present Tense

Stylists recommend the use of the present tense [10,12], as it is more direct and deemed easier to understand for non-native speakers. We assessed this by ascertaining the ratio of (present tense)/(present + past tense) (R4).

## Rule 5: Avoid Adjectives and Adverbs

Using few adjectives and adverbs avoids fluff and keeps the text short and easy to understand [4,8,9,12]. We measured the effect of having a proportion of adjectives and adverbs smaller than that typical for the journal (R5).

## Rule 6: Focus

Many authors suggest sticking to a single point, and reiterating the “take home” message [5,6,11,13,14]. We captured this with the proportion of words in the abstract that were also keywords (R6).

## Rule 7: Signal Novelty and Importance

There is conflicting advice on whether to explicitly state the significance of your work. Stressing that the work is novel and solves important problems helps to “sell” the article [12,15]. Opponents of this rule say that all published work should already meet these criteria [8,13]. We examined this by checking whether the abstract contained at least one word signaling novelty (e.g., “novel,” “new,” “innovative” [R7a]) and, separately, a word signaling importance (e.g., “key,” “significant,” “crucial” [R7b]).

## Rule 8: Be Bold

Many authors suggest “selling” the work forcefully and stressing positive results. We tested this by measuring the ratio (superlatives)/(superlatives + comparatives) (R8).

## Rule 9: Show Confidence

Similarly, using too many “hedge words” (e.g., “somewhat,” “speculative,” “appear,” “almost,” “largely”) can signal a lack of confidence in the work. We explored this with the measure of fewer hedge words in the abstract (R9).

## Rule 10: Avoid Evocative Words

A style perceived as too flowery or involving the overuse of highly evocative words is discouraged. We tested whether using words perceived as “pleasant,” “active,” or “easy to imagine” led to more citations than those for abstract containing “unpleasant,” “passive,” or “hard to imagine” words [16–18] (R10a–c).

## Results

**Fig 1:**
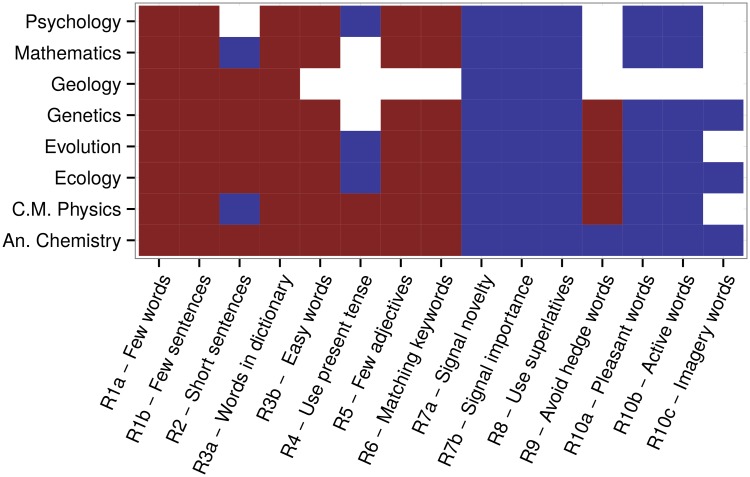
Effect of abstract features on citations. For each discipline (rows) and each abstract feature (columns), we measured whether a certain feature (e.g., having fewer words than the typical abstract published in the same journal [R1a]) led to a significant increase (blue) or decrease (red) in total citations. We considered an effect positive or negative only if the associated probability of being zero was smaller than 0.01/15 (i.e., we applied the Bonferroni correction to obtain an overall significance level of 1%).

In Fig 1, we report the sign of the effect associated with each abstract feature (column) for each discipline (row). Surprisingly, half of the typical suggestions—including those that are most common, about brevity and clarity—are associated with a significant decrease in citations.

We find that shorter abstracts (fewer words [R1a] and fewer sentences [R1b]) consistently lead to fewer citations, with short sentences (R2) being beneficial only in Mathematics and Physics. Similarly, using more (rather than fewer) adjectives and adverbs is beneficial (R5). Also, writing an abstract with fewer common (R3a) or easy (R3b) words results in more citations.

The use of the present tense (R4) is beneficial in Biology and Psychology, while it has a negative impact in Chemistry and Physics, possibly reflecting differences in disciplinary culture.

While matching the keywords (R6) leads to universally negative outcomes, signaling the novelty and importance of the work (R7) has positive effects. The use of superlatives (R8) is also positive, while avoiding “hedge” words is negative in Biology and Physics, but positive in Chemistry.

Finally, choosing “pleasant,” “active,” and “easy to imagine” words (R10) has positive effects across the board.

**Fig 2:**
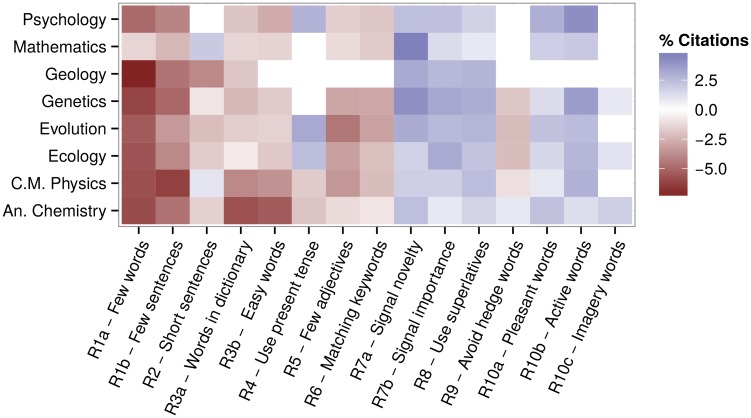
Size of the effects. Same designations as Fig 1, but measuring the benefit/cost of having a certain feature one standard deviation above the mean for the corresponding journal.

When we measured effect sizes (Fig 2), we found that abstract features can have a strong influence on citations. Being one standard deviation above the mean for a given feature (with respect to the mean for corresponding journal) can increase citations by 4.6% (Mathematics [R7a]), or decrease them by 7.2% (Geology [R1a]). When analyzing each journal separately, we find qualitatively the same results (S3–S10 Figs).

## Conclusions

We have found that—when it comes to abstracts—“more is more,” despite clear and abundant advice to the contrary.

This is an interesting and surprising result. An intriguing hypothesis is that scientists have different preferences for what they would like to read versus what they are going to cite. Despite the fact that anybody in their right mind would prefer to read short, simple, and well-written prose with few abstruse terms, when building an argument and writing a paper, the limiting step is the ability to find the right article. For this, scientists rely heavily on search techniques, especially search engines, where longer and more specific abstracts are favored. Longer, more detailed, prolix prose is simply more available for search. This likely explains our results, and suggests the new landscape of linguistic fitness in 21st century science. Future studies could investigate the relationship between stylistic features and retrievability directly, as well as the strength of the relationship between retrievability and citation performance.

Another interesting finding is that there is very little variation across disciplines, with only three out of fifteen features displaying sign changes among the diverse fields we examined.

Scientists are skeptical by disposition, and this exercise shows that, rather than taking advice at face value, they can apply the same machinery they use to interrogate nature to put these recommendations to the test—and write a lengthy, convoluted, highly-indexible, self-describing abstract.

## Supporting Information

S1 Text Supporting Methods and Results. Description of the data, the features analyzed and the statistical models; discipline-specific results.
(PDF)

S1 Fig Distribution of citations through time. Figure showing that citations received by the articles in a journal/year combination are approximately log-normally distributed.
(TIFF)

S2 Fig Number of words in abstracts. Distribution of the number of words in the abstract divided by discipline.
(TIFF)

S3 Fig Effect sizes in Analytical Chemistry. As Fig 2, but analyzing Analytical Chemistry journals.
(TIFF)

S4 Fig Effect sizes in Ecology. As Fig 2, but analyzing Ecology journals.
(TIFF)

S5 Fig Effect sizes in Evolution. As Fig 2, but analyzing Evolution journals.
(TIFF)

S6 Fig Effect sizes in Genetics. As Fig 2, but analyzing Genetics journals.
(TIFF)

S7 Fig Effect sizes in Geology. As Fig 2, but analyzing Geology journals.
(TIFF)

S8 Fig Effect sizes in Mathematics. As Fig 2, but analyzing Mathematics journals.
(TIFF)

S9 Fig Effect sizes in Condensed Matter Physics. As Fig 2, but analyzing Condensed Matter Physics journals.
(TIFF)

S10 Fig Effect sizes in Psychology. As Fig 2, but analyzing Psychology journals.
(TIFF)
