# Supplementary material for: Ten Simple (Empirical) Rules for Writing Science
Source: PLoS Comput Biol. 2015 Apr 30;11(4):e1004205. doi: 10.1371/journal.pcbi.1004205 (PMC4415812; doi:10.1371/journal.pcbi.1004205)
Supplement: S1 Text — Description of the data, the features analyzed and the statistical models; discipline-specific results. (PDF) [file pcbi.1004205.s001.pdf]

Ten Simple (Empirical) Rules for Writing Science Supporting Information

Cody J. Weinberger[1], James A. Evans[2,3], Stefano Allesina[1,3,*]

1 Department of Ecology & Evolution, University of Chicago, Chicago, IL, USA
2 Department of Sociology, University of Chicago, Chicago, IL, USA
3 Computation Institute, University of Chicago, Chicago, IL, USA

*** sallesina@uchicago.edu**

S1 Text: Materials and Methods

Data

The data was downloaded from Scopus (`scopus.com`) in May 2013. For each journal, we downloaded all the records marked as "Article" in the database (i.e., we excluded the categories "Editorial", "Erratum", "Letter", "Note","Review", etc.). In this way, we guaranteed that the downloaded records were original research articles, and not other types of documents.

Table 1. Disciplines

ISI JCR category	Abbreviation
CHEMISTRY, ANALYTICAL	An. Chemistry
ECOLOGY	Ecology
EVOLUTIONARY BIOLOGY	Evolution
GENETICS & HEREDITY	Genetics
GEOLOGY	Geology
MATHEMATICS	Mathematics
PHYSICS, CONDENSED MATTER	C.M. Physics
PSYCHOLOGY	Psychology

The eight disciplines analyzed in this work. Left: the category reported by the ISI Journal of Citation Reports. Right: the abbreviation used in tables and figures.

We chose eight disciplines according to the categorization made by the ISI Journal of Citation Reports (`thomsonreuters.com/journal-citation-reports/`). In S1 Table, we report the name of the category as well as the abbreviation we used throughout this work. For each of the eight disciplines, we downloaded from Scopus information on all the articles published from 1996 to 2012, included. For each article, we recorded the number of citations, authors, references, words in the abstract, as well as the year of publication, the journal title, the discipline, the full set of keywords, and, naturally, the abstract. The disciplines were chosen so that biology was represented by three closely-related fields (Ecology, Evolution, Genetics), and the "outgroup"

contained a wide variety of fields. Some journals belong to multiple disciplines (e.g., the journal "Evolution" is considered in Ecology, in Evolution, and in Genetics).

To make sure that all records were complete, and that the abstracts were correctly recorded (sometimes Scopus reported articles in which the abstract was "[No abstract available]" or similar), we excluded all the articles for which: a) the abstract had less than 50 words; b) we found no author; c) we found less than ten references. This left us with 1,065,139 records with three disciplines being much smaller (Geology: 24,475 records; Evolution: 56,188; Psychology: 56,417) than the others (Mathematics: 133,644; Ecology: 164,287; Genetics: 190,525; An. Chemistry: 195,464; C.M. Physics: 244,139).

Analysis

The goal of the analysis is to ascertain the effect of a particular abstract feature, x, on the number of citations an article receives. To this end, we want to account for factors that are likely to influence citation counts, such as the journal where the article has been published, the age of the article, its number of authors and number of references.

First, instead of modeling citation counts, we chose $\log(\text{citations} + 1)$ as our response variable. In this way, given that citations to articles of a given age tend to follow a log-normal distribution [1], we should recover approximately a normal distribution for each journal-year combination. In Fig. 1, we show that a normal distribution well-approximates the $\log(\text{citations} + 1)$, especially for the older articles. Fig. 1 also shows that the mean of $\log(\text{citations} + 1)$ changes non-linearly with time. Because of this fact, we treated each journal-year combination as a different categorical variable in our regression analysis. This effectively removes the time-dependence of citation counts.

Figure 1. Distribution of citations through time. Histogram (bars) and smoothed density (blue curve) for the $\log(\text{citations} + 1)$ for the journal *Ecology* (left) and *Physical Reviews B* (right). Only the even years are presented. For the earlier years, the histogram is well-approximated by a normal distribution.

Most journals have strict requirements on the number of words in the abstract, the number of references, and sometimes even the number of authors. Moreover, these quantities vary widely between disciplines, as shown by Fig. 2. Therefore, instead of using the raw measures we recorded, in the regressions we used their z-scores: take $(\text{n. words})_i$ to be the number of words in the abstract of article i published in journal $j(i)$ in year $y(i)$. Then, we took $z(\text{n. words})_i = (\text{n. words})_i - \mu(\text{n. words})_{j(i)})/\sigma(\text{n. words})_{j(i)}$, where $\mu(\text{n. words})_{j(i)}$ is the average number of words in the abstracts published in journal $j(i)$ (considering all years) and $\sigma(\text{n. words})_{j(i)}$ is the corresponding standard deviation.

Figure 2. Number of words in abstracts. Violin plots showing the distribution of the number of words in the abstracts, divided by discipline. While in Mathematics and C.M. Physics all journals seem to have adopted a similar length requirement, the distributions for several other disciplines display multi-modality, due to the fact that different journals have different requirements. Notably, all disciplines contain outlier articles with extremely lengthy abstracts, often exceeding 1000 words (e.g., Psychology: [2] > 1600 words, Ecology: [3] ≈ 1500 words).

With this notation in place, we can write the linear model:

$$\log(\text{citations} + 1)_i = \alpha + \beta_{j(i)y(i)} + \gamma z(\text{n. authors})_i + \delta z(\text{n. refs.})_i + \zeta z(x)_i + \epsilon_i \quad (1)$$

where α is a common intercept, $\beta_{j(i)y(i)}$ specifies the effect of journal-year combination, γ measures the effect of having a number of authors that is larger than the mean for the journal, δ the effect of having more references than what is typical for the journal, and ζ measures the effect of having a certain feature of the abstract, x, with values that are above the mean for the journal. The residuals are stored in ϵ_i.

Note that ζ measures the effect of being one standard deviation above the mean for the journal. Suppose that article a has a feature x (e.g., number of words) taking exactly the value of the mean for the corresponding journal. Then $z(x)_a = 0$. Article b has the same features as a, besides having x exactly one standard deviation above the mean. Thus, $z(x)_b = 1$. The difference $\log(\text{citations} + 1)_b - \log(\text{citations} + 1)_a = \zeta$. Exponentiating, we obtain:
$e^{\zeta} = (\text{citations} + 1)_b/(\text{citations} + 1)_a \approx (\text{citations})_b/(\text{citations})_a$. Hence, $(e^{\zeta} - 1) \cdot 100$ is the percentage of citations gained or lost due to having feature x one standard deviation above the mean.

We ran a different regression (using the package `biglm` of the statistical software `R`) for each discipline, and then repeated the analysis at the journal level. Basically, we are interested in the sign and magnitude of ζ for each feature of the abstract x and each discipline. For simplicity, we tested each feature of the abstract separately, rather than trying to model them all together. Notice that many features are correlated (e.g., it is difficult to write an abstract with many sentences but few words), so that correlated features will tend to return similar effects.

Because we are testing multiple hypotheses using the same data set, we used the Bonferroni correction when determining whether ζ is significantly different from 0. We used a desired significance level of 0.01 when analyzing disciplines (for which we have tens of thousand of records), and 0.05 for journals (for which we have much less data). These are extremely conservative criteria, especially for the case of journals, where we have limited statistical power.

Abstract Features

Here we detail how the measures illustrated in the main text were calculated.

R1. We measured the total number of words (R1a), and total number of sentences (R1b). Words and sentences were identified using the library Natural Language Tool Kit (`nltk`) [4, 5] for `python`. The explanatory variables were taken to be $-z(\text{num. words})_i$ and $-z(\text{num. sentences})_i$, as the advice is to keep the abstract short.

R2. We measured the mean number of words per sentence. Words and sentences were again identified using `nltk`. The explanatory variable is $-z(\text{avg. words per sentences})_i$.

R3. We measured the proportion of unique words in the abstract that are found in the *GNU Aspell* dictionary (R2a), or in a list of 2954 words taken from the Dale-Chall list of Easy Words (R2b).

R4. We tagged all verbs using `nltk`, and computed the fraction (present + gerund) / (present + gerund + past + past participle).

R5. We tagged all words using `nltk` and calculated (adjectives + adverbs) / (total words).

R6. We counted how many words in the abstract were also keywords (when keywords were reported; otherwise we set this value to not available).

R7. We set the variable to 1 whenever the abstract contained at least a word signaling novelty (R7a) or importance (R7b) and to zero otherwise.

R8. We used `nltk` to compute the proportion (superlatives) / (superlatives + comparatives).

R9. We computed the proportion of words in the abstracts that were in a dictionary of "hedge words".

R10. We scored each word in the abstract using the Whissell's Dictionary of Affective Language [6–8] (taking a value of 0 when the word was not found in the dictionary), summed the values, and divided by the number of words.

S2 Text: Supporting Results

Fig. 3-10 show the sign and magnitude of the effects at the journal level. We performed a regression for each journal within each discipline. Notably, although the power is much reduced due to the limited size of the data, the signs of interactions are largely consistent with what found at the discipline level.

Figure 3. Effect sizes in An. Chemistry. As in main text Fig. 2, but performing a regression for each journal.

Figure 4. Effect sizes in Ecology. As in main text Fig. 2, but performing a regression for each journal.

Figure 5. Effect sizes in Evolution. As in main text Fig. 2, but performing a regression for each journal.

Figure 6. Effect sizes in Genetics. As in main text Fig. 2, but performing a regression for each journal.

Figure 7. Effect sizes in Geology. As in main text Fig. 2, but performing a regression for each journal.

Figure 8. Effect sizes in Mathematics. As in main text Fig. 2, but performing a regression for each journal.

Figure 9. Effect sizes in C.M. Physics. As in main text Fig. 2, but performing a regression for each journal.

Figure 10. Effect sizes in Psychology. As in main text Fig. 2, but performing a regression for each journal.

References

1. Radicchi F, Fortunato S, Castellano C. Universality of citation distributions: Toward an objective measure of scientific impact. Proceedings of the National Academy of Sciences. 2008;105(45):17268–17272.
2. Mangeney-Hirsch S, Courtois R, Lecocq G, Rusch E, Porcheron E; Elsevier. Abus sexuel et inaptitude à l'éducation physique et sportive. Annales Médico-psychologiques, revue psychiatrique. 2008;166(10):799–807.
3. Landman NH, Kennedy WJ, Cobban WA, Larson NL. Scaphites of the "nodosus group" from the Upper Cretaceous (Campanian) of the Western Interior of North America. Bulletin of the American Museum of Natural History. 2010;p. 1–242.
4. Loper E, Bird S. NLTK: The natural language toolkit. In: Proceedings of the ACL-02 Workshop on Effective tools and methodologies for teaching natural language processing and computational linguistics-Volume 1. Association for Computational Linguistics; 2002. p. 63–70.
5. Bird S, Klein E, Loper E. Natural language processing with Python. O'Reilly Media, Inc.; 2009.
6. Sweeney K, Whissell C. A dictionary of affect in language: I. Establishment and preliminary validation. Perceptual and motor skills. 1984;59(3):695–698.
7. Whissell C. The dictionary of affect in language. Emotion: Theory, research, and experience. 1989;4(113-131):94.
8. Whissell C. Using the revised dictionary of affect in language to quantify the emotional undertones of samples of natural language 1, 2. Psychological reports. 2009;105(2):509–521.
