# Supplementary figures and images for: Ten Simple (Empirical) Rules for Writing Science
Source: PLoS Comput Biol. 2015 Apr 30;11(4):e1004205. doi: 10.1371/journal.pcbi.1004205 (PMC4415812; doi:10.1371/journal.pcbi.1004205)

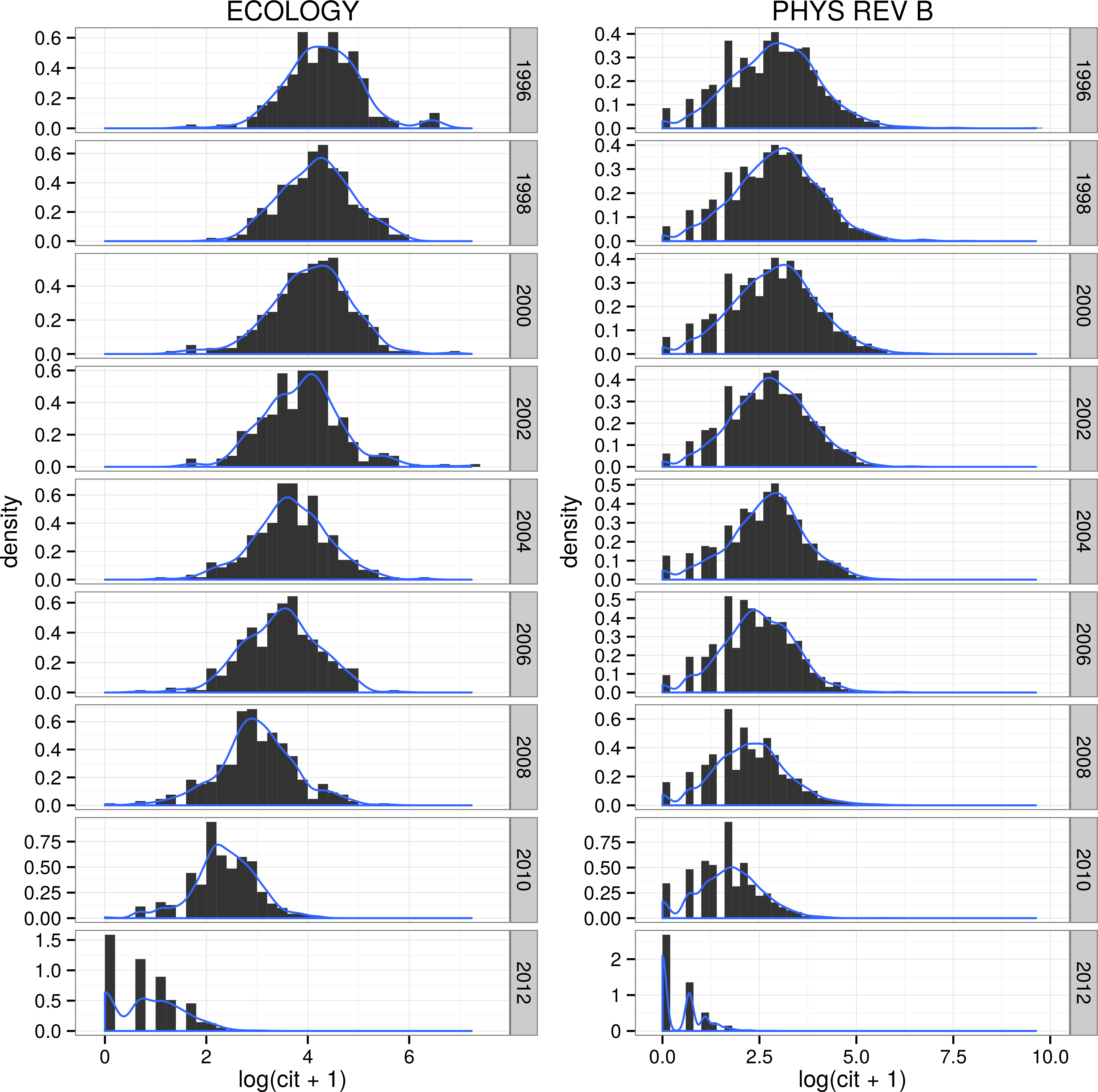

Supplement: S1 Fig — Figure showing that citations received by the articles in a journal/year combination are approximately log-normally distributed. (TIFF) [file pcbi.1004205.s002.tiff]

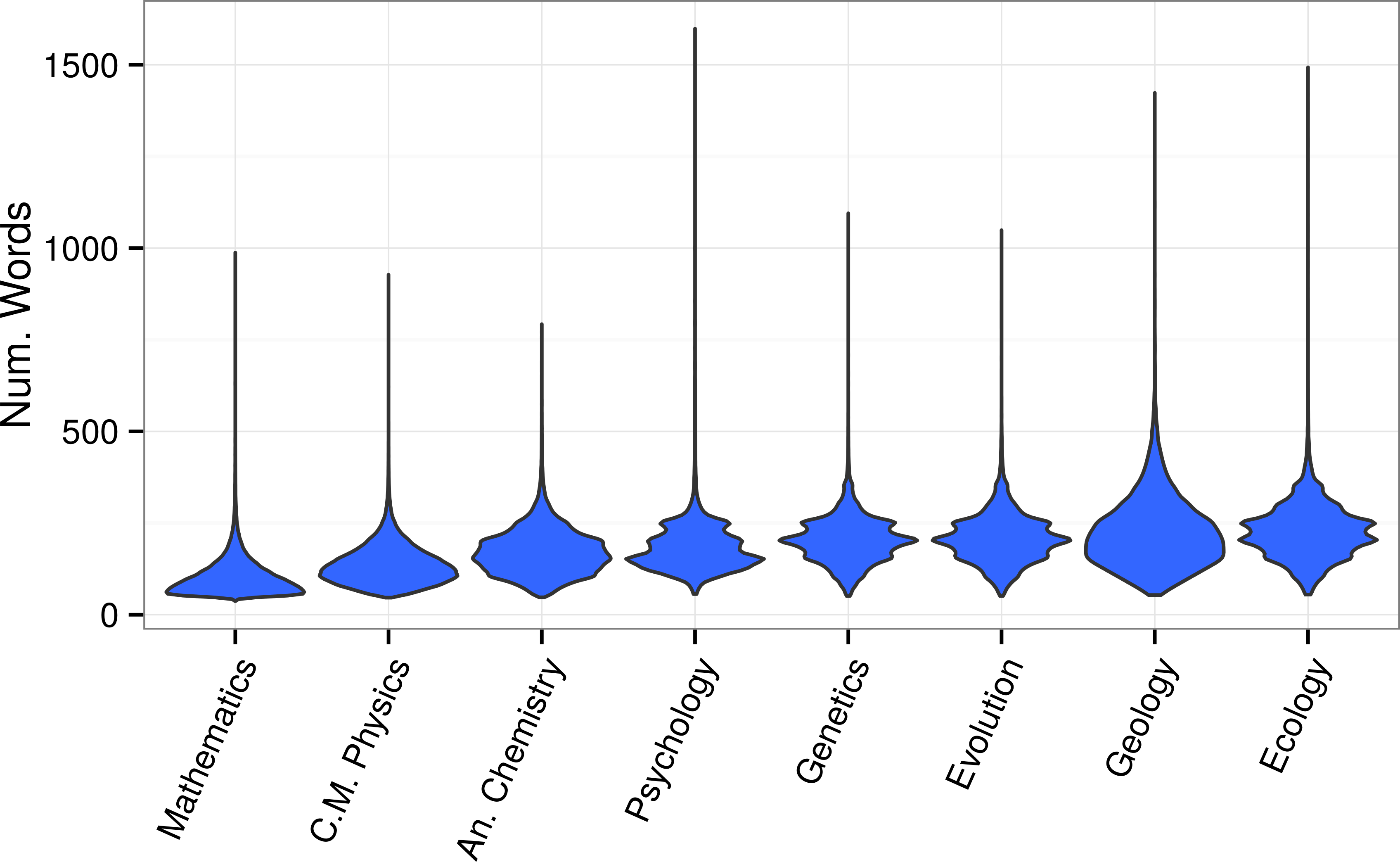

Supplement: S2 Fig — Distribution of the number of words in the abstract divided by discipline. (TIFF) [file pcbi.1004205.s003.tiff]

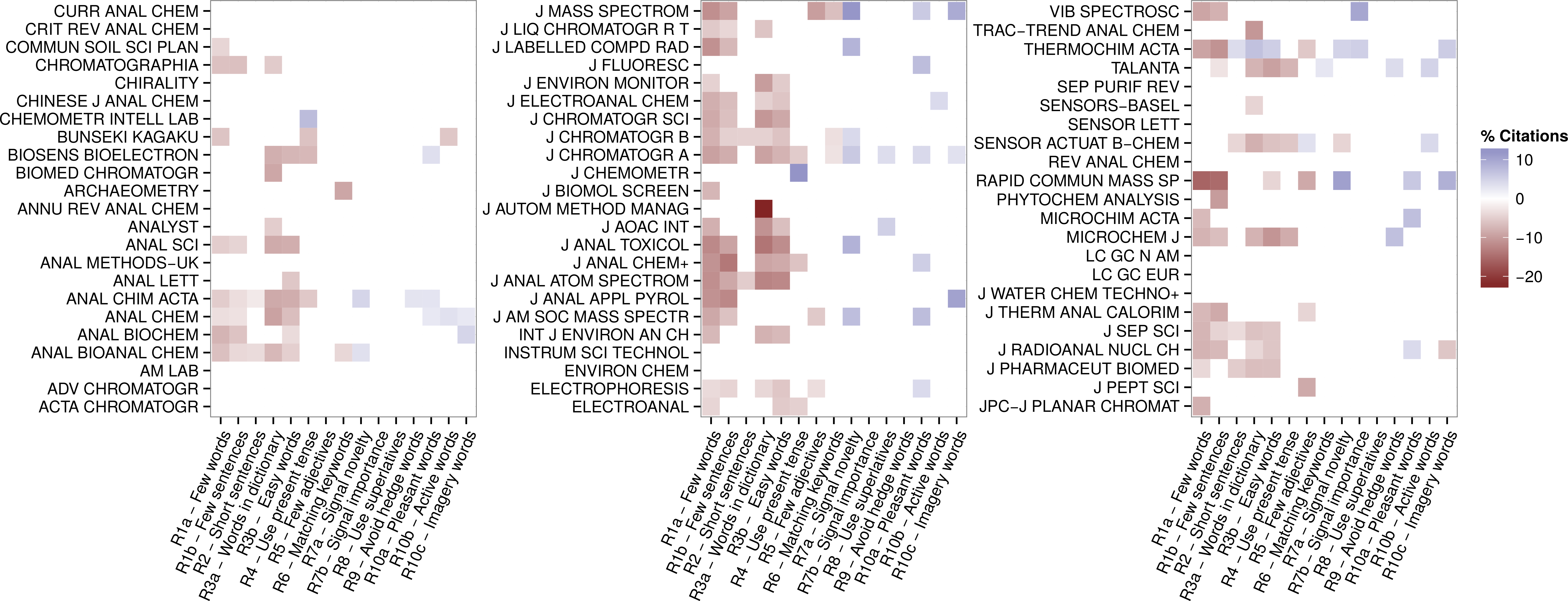

Supplement: S3 Fig — As Fig 2, but analyzing Analytical Chemistry journals. (TIFF) [file pcbi.1004205.s004.tiff]

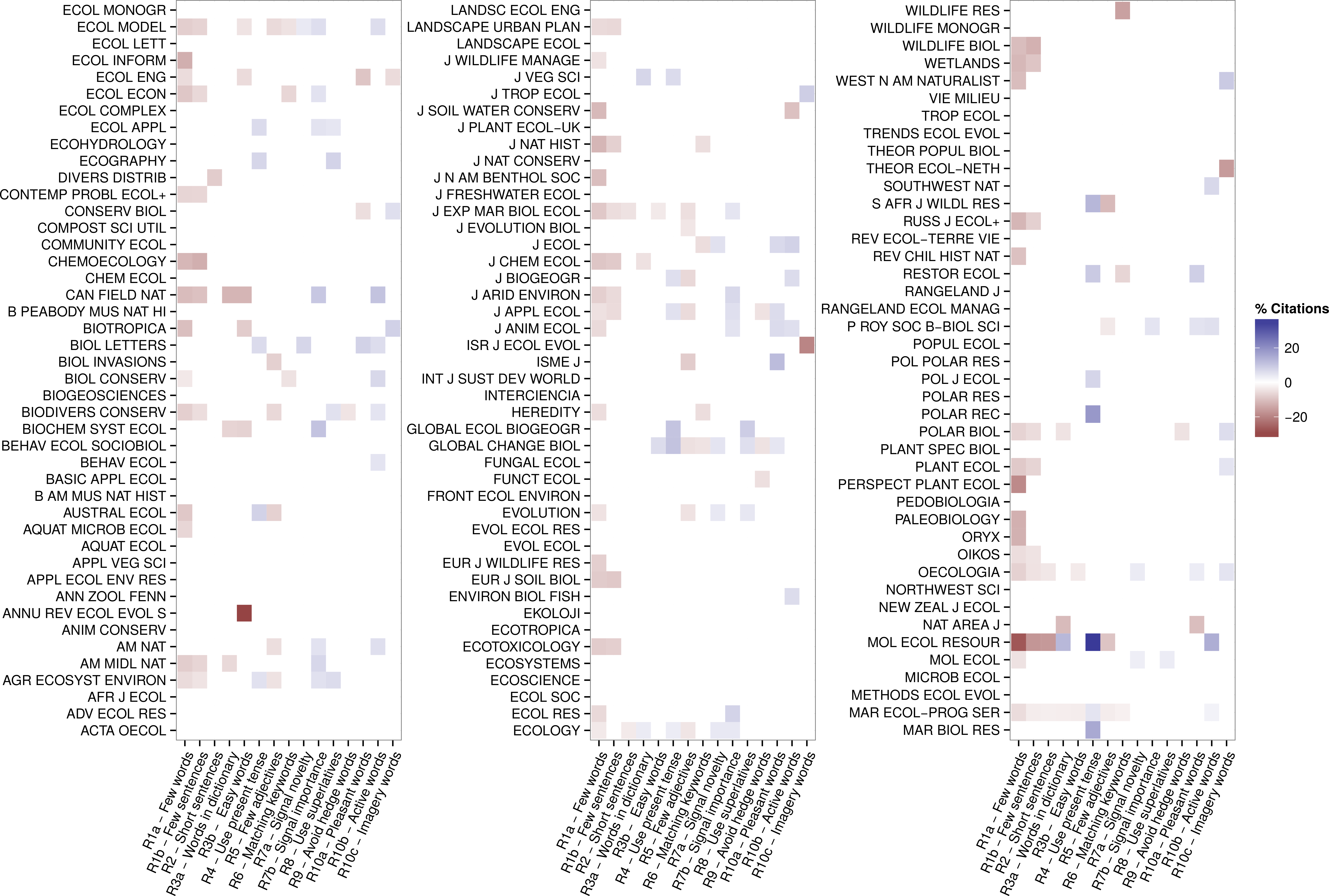

Supplement: S4 Fig — As Fig 2, but analyzing Ecology journals. (TIFF) [file pcbi.1004205.s005.tiff]

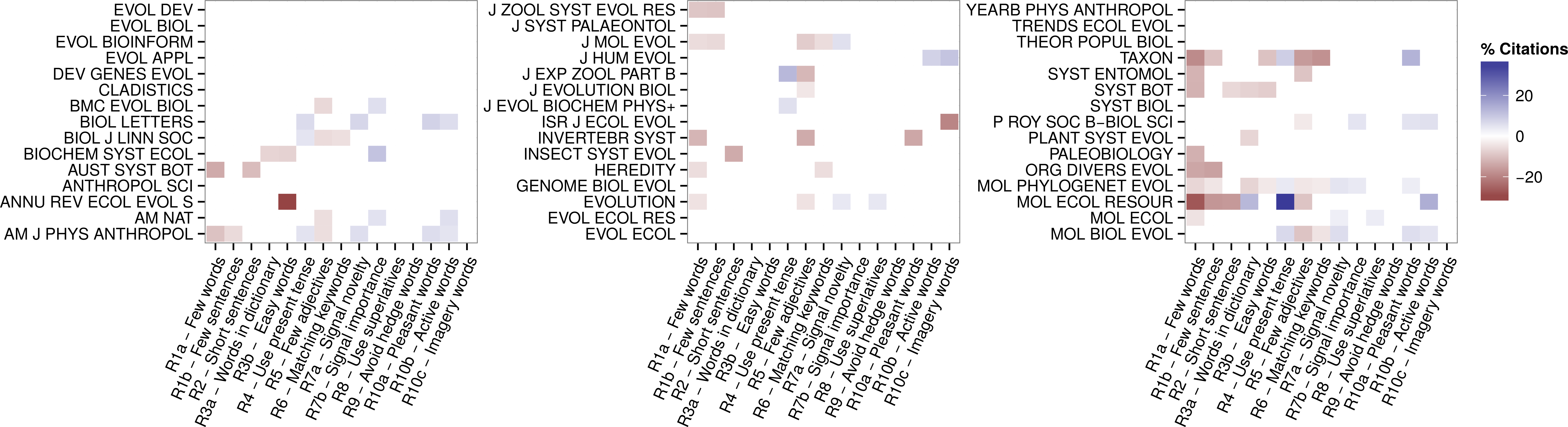

Supplement: S5 Fig — As Fig 2, but analyzing Evolution journals. (TIFF) [file pcbi.1004205.s006.tiff]

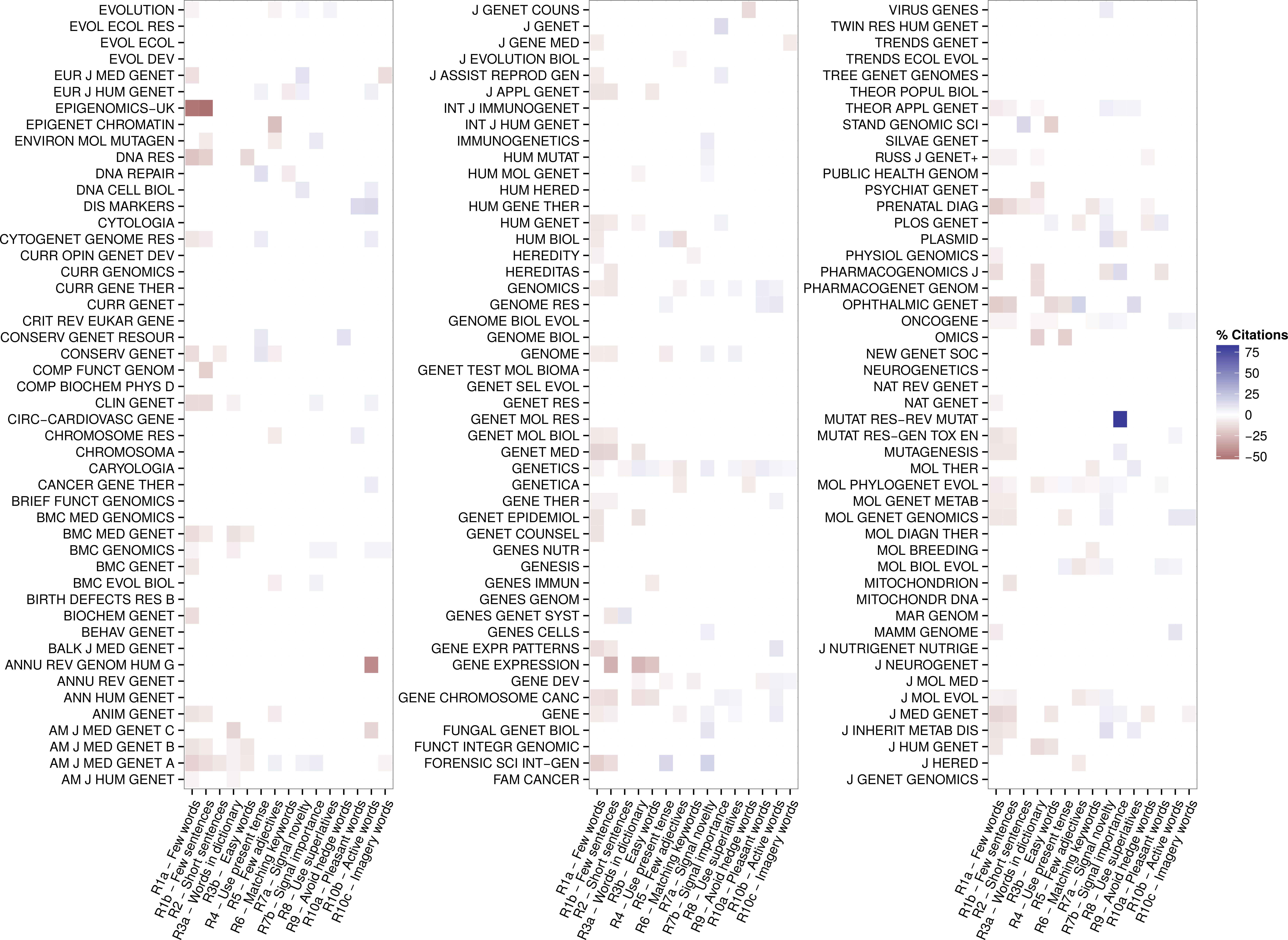

Supplement: S6 Fig — As Fig 2, but analyzing Genetics journals. (TIFF) [file pcbi.1004205.s007.tiff]

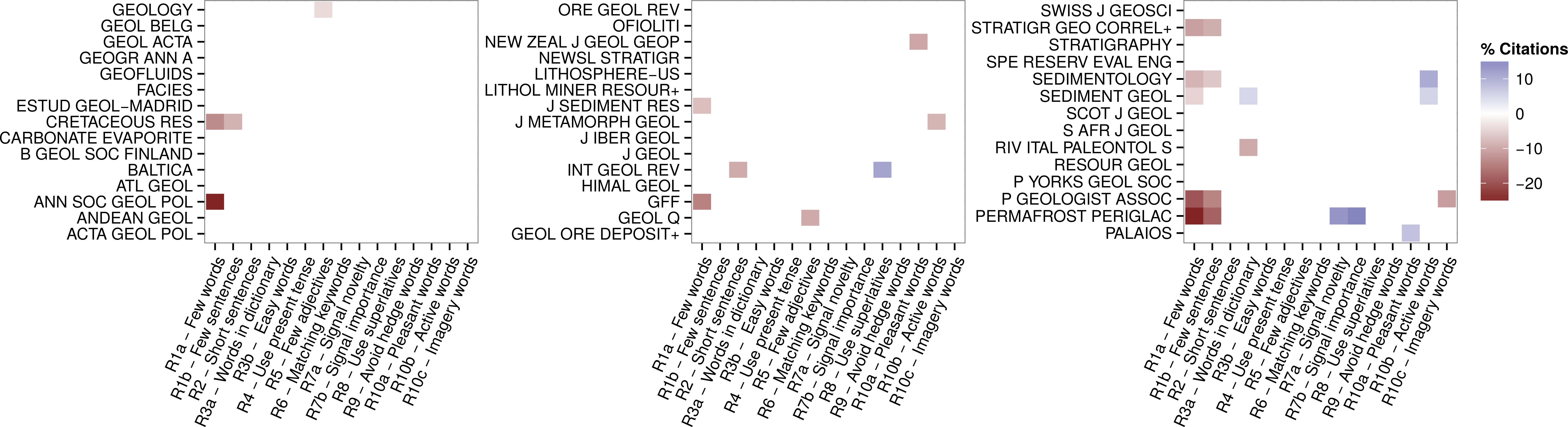

Supplement: S7 Fig — As Fig 2, but analyzing Geology journals. (TIFF) [file pcbi.1004205.s008.tiff]

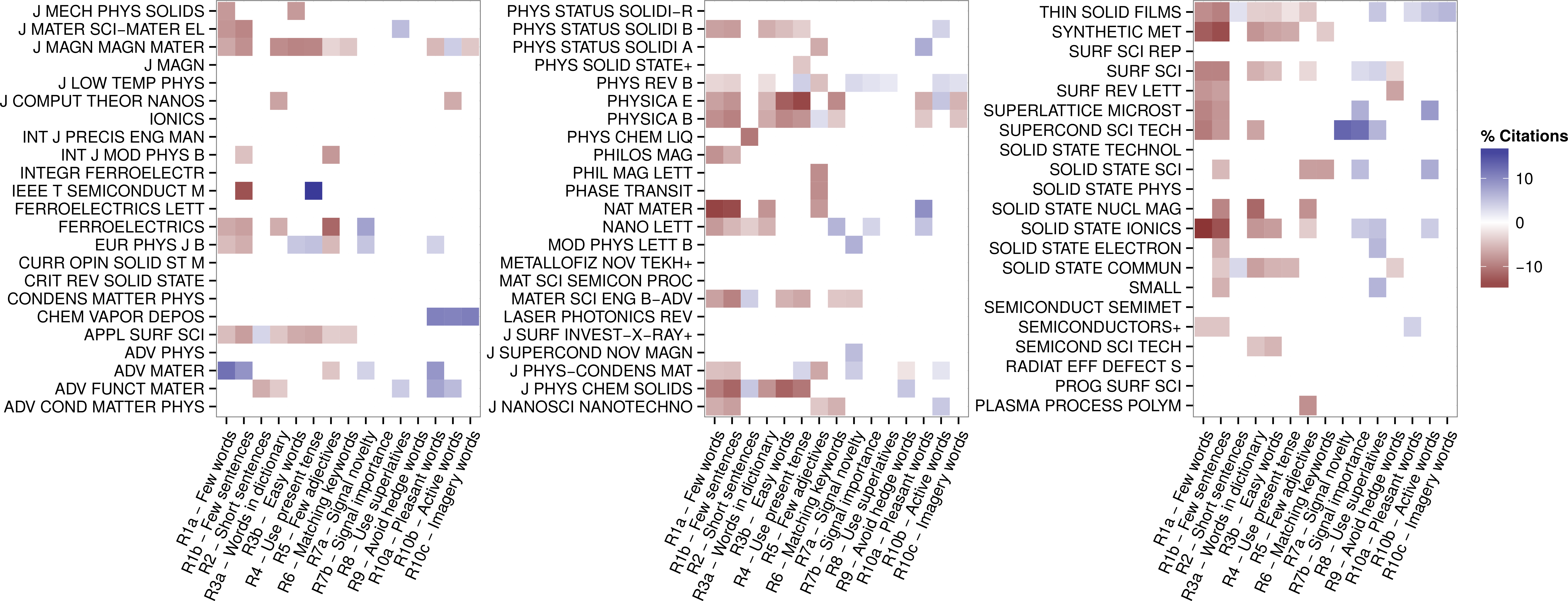

Supplement: S9 Fig — As Fig 2, but analyzing Condensed Matter Physics journals. (TIFF) [file pcbi.1004205.s010.tiff]

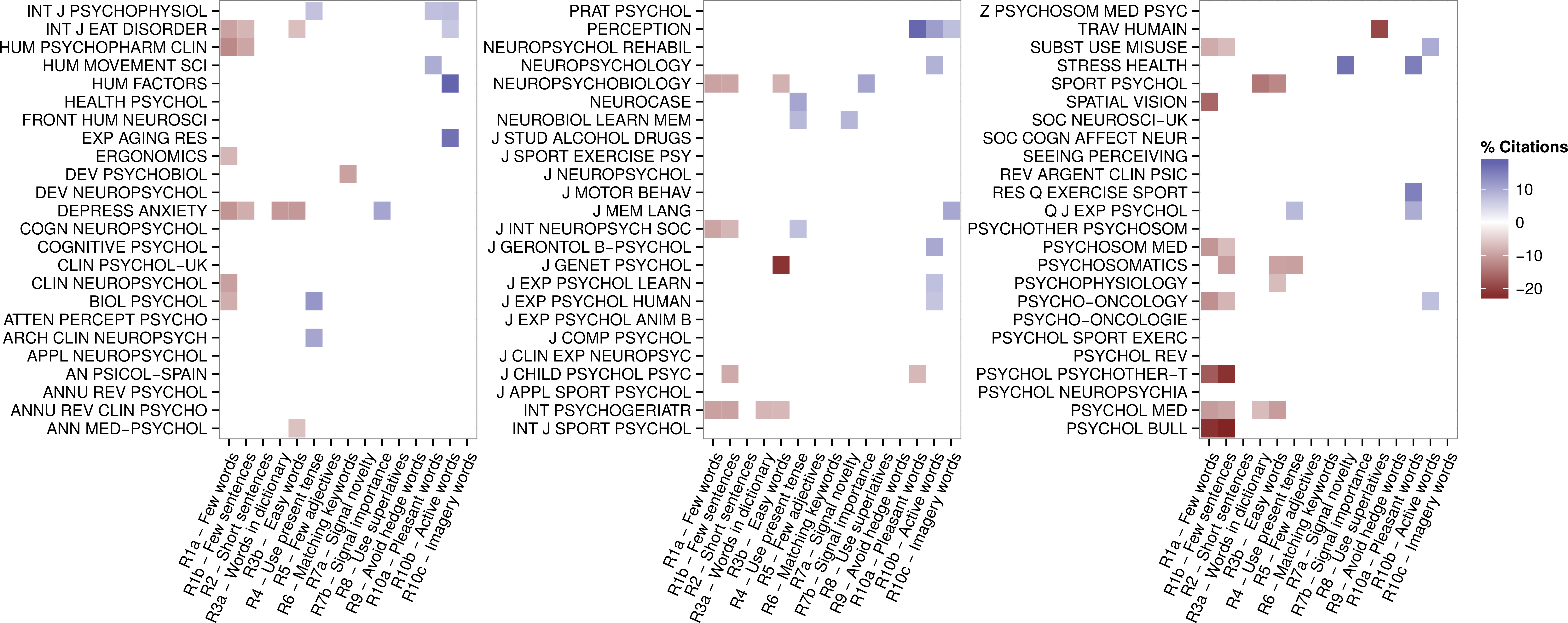

Supplement: S10 Fig — As Fig 2, but analyzing Psychology journals. (TIFF) [file pcbi.1004205.s011.tiff]
